# IL-6 and Olfactory Dysfunction: Focus on Changes, Effects, and Mechanisms

**DOI:** 10.1155/mi/5891188

**Published:** 2025-05-19

**Authors:** Xiao-Yu Song, Ya-Kui Mou, Han-Rui Wang, Yao Wang, Wan-Chen Liu, Ting Yang, Cai-Yu Sun, Chao Ren, Xi-Cheng Song

**Affiliations:** ^1^Department of Otorhinolaryngology, Head and Neck Surgery, Yantai Yuhuangding Hospital, Qingdao University, Yantai, China; ^2^Shandong Provincial Key Laboratory of Neuroimmune Interaction and Regulation, Yantai Yuhuangding Hospital, Yantai, China; ^3^Shandong Provincial Clinical Research Center for Otorhinolaryngologic Diseases, Yantai Yuhuangding Hospital, Yantai, China; ^4^Yantai Key Laboratory of Otorhinolaryngologic Diseases, Yantai Yuhuangding Hospital, Qingdao University, Yantai, China; ^5^Department of Neurology, Yantai Yuhuangding Hospital, Qingdao University, Yantai, China

**Keywords:** biomarkers, IL-6, inflammation, mechanism, olfactory dysfunction

## Abstract

The sense of smell is vital for human life and risk identification. Many diseases can cause olfactory disorders, and early identification and intervention of olfactory disorders are crucial. Currently, the diagnosis of olfactory disorders in clinical practice mostly relies on subjective visual analog scale (VAS) evaluations, expensive and complex imaging, and neurophysiological examinations, which lead to poor patient compliance and low completion rates. Therefore, there is an urgent need to identify novel, objective, easily detectable biological indicators. Interleukin-6 (IL-6) is an inflammatory factor that is closely associated with olfactory dysfunction in various diseases. However, the role of IL-6 in the occurrence and development of olfactory disorders is not yet clear, which limits its clinical application. This article reviews the changes and possible mechanisms of IL-6 in various diseases associated with olfactory disorders, with the aim of providing a reference for the clinical application of IL-6 as a biomarker for olfactory disorders and promoting an in-depth exploration of its mechanism in the occurrence and development of olfactory disorders.

## 1. Background

Smells are important sensation in daily life. They not only bring joy during eating, but also cause discomfort when smelling foul odors. The smell of burning and food spoilage can help avoid dangerous situations, such as fire and food poisoning. Therefore, when olfactory disorders occur, they not only reduce the quality of life of patients, but may also pose a threat to their lives [1, 2]. Olfactory dysfunction (OD) refers to the abnormal perception of odors caused by qualitative and/or functional lesions in various links of the olfactory pathway during odor perception, transmission, and integration of information analysis. OD includes both quantitative and qualitative olfactory impairment. The former includes decreased sense of smell, loss of smell, and olfactory hypersensitivity, whereas the latter includes olfactory inversion and hallucinations [3]. In the traditional classification, OD is divided into two categories: conductive and neuropathic. Conductive OD is typically caused by diseases or injuries to the nasal cavity, throat, and other areas, such as sinusitis and nasal polyps, these diseases affect the transmission of odor molecules to olfactory cells, whereas neuropathic OD is mostly caused by central nervous system lesions, such as head trauma and Parkinson's disease (PD). These lesions affect processing and recognition of olfactory signals [4–6]. In recent years, it has been suggested that OD should no longer be classified as conductive or neuropathic because chronic sinusitis with OD after infection often involves both components. Therefore, the latest classification shows that similar to age-related functional impairments, OD should be classified as congenital and acquired [7–9]. The incidence of OD in the population reaches up to 40%; however, current attention to OD is still insufficient, which is mainly attributed to the lack of objective, effective, and convenient methods for OD identification. At present, the olfactory psychophysical tests commonly used in clinical practice, including subjective visual analog scale (VAS) evaluation and expensive and complex imaging and neurophysiological examinations, have poor patient cooperation and low completion rates. The recognition rate of OD detected by the above methods is only 27% [10]. Therefore, exploring simpler and more efficient methods is currently an important direction in OD research with high expectations for hematological markers. Interleukin-6 (IL-6) has been used as an inflammatory marker in the clinical evaluation of patients with inflammation, infections, or immune disorders. Several studies have reported that IL-6 expression is associated with OD in various diseases (Table 1 [11–15]). However, the role of IL-6 in the occurrence and development of OD remains unclear, which affects its clinical application. This article reviews the characteristics and possible mechanisms of IL-6 changes in various diseases associated with OD to provide evidence for the clinical application of IL-6 as a biomarker of OD and to promote in-depth exploration of its mechanism of action in the occurrence and development of OD.

## 2. Introduction to IL-6

**Figure 1:**
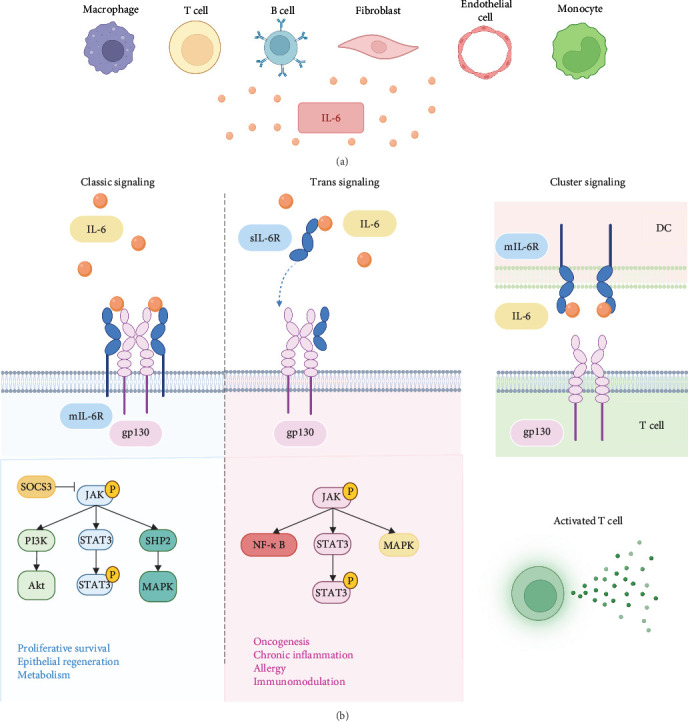
The main sources and signaling pathways of interleukin 6. (A) schematic diagram illustrating the primary cellular sources of IL-6, including monocytes, macrophages, T cells, B cells, fibroblast, and endothelial cells. (B) depiction of the three major IL-6 signaling pathways. IL-6 binds to the mIL-6R and activates the classical signaling pathway, which exerts anti-inflammatory effects. Additionally, IL-6 can bind to the sIL-6R, triggering the trans-signaling pathway that promotes inflammation. Through cluster signaling, DCs are able to trans-present IL-6 through a complex containing DC-expressed IL-6R*α* bound to IL-6 that can interact with gp130 expressed on T cells, mediating pathogenic T cell differentiation (The figure is created in https://BioRender.com).

IL-6 is a typical member of the IL-6 cytokine family, which consists of 10 members, including IL-6, interleukin-11 (IL-11), interleukin-27 (IL-27), oncostatin M (OSM), leukemia inhibitory factor (LIF), ciliary neurotrophic factor (CNTF), and cardiotrophin-1 (CT-1). Otropin-like cytokine factor 1 (CLCF1), interleukin-35 (IL-35), and interleukin-39 (IL-39) have also been identified [16]. IL-6 is a single-chain glycoprotein with a relative molecular mass of 21–30 kD containing 212 amino acid residues, and its gene is localized on chromosome 7. IL-6 is an inflammatory factor with two types of receptors: the membrane receptor mIL-6R, which is expressed on the cell membrane surface, and the soluble receptor sIL-6R, which is present in the circulatory system. They cannot transduce intracellular signals on their own and require assistance from the transmembrane protein gp130 (CD130) [17–19]. IL-6 has both pro-inflammatory and anti-inflammatory properties and acts via different signaling pathways. Within the classic signaling pathway, IL-6 binds to mIL-6R and forms a high-affinity IL-6/mIL-6R/gp130 complex with gp130. Soluble IL-6 receptor (sIL-6R) can be generated by the proteases ADAM17 (a disintegrin and metalloproteinase 17), and then IL-6 binds to sIL-6R in circulation and further forms the IL-6/sIL-6R/gp130 complex with gp130 [20, 21]. Both IL-6/mIL-6R/gp130 and IL-6/sIL-6R/gp130 complexes can activate the JAK pathway, leading to tyrosine residue phosphorylation on gp130. However, the IL-6/mIL-6R/gp130 complex recruits and phosphorylates Signal Transducers and Activators of Transcription 3 (STAT3), which then forms dimers, moves to the nucleus, and promotes suppressor of cytokine signaling 3(SOCS3) transcription. SOCS3 directly inhibits JAK kinases, thereby preventing further phosphorylation of STAT proteins [22]. In addition to activating the JAK/STAT pathway, phosphatase SHP-2 is recruited to tyrosine-phosphorylated gp130 and further phosphorylated by JAK1, mediating the Ras-Raf-MAPK signaling involved in cell proliferation and differentiation. This process regulates ERK1/2, promoting transcription factors for anti-inflammatory cytokine expression, while SHP2 and the protein inhibitor of activated STAT (PIAS) can help alleviate inflammation and promote tissue repair [23, 24]. The PI3K/Akt (phosphatidylinositide-3-kinase) signaling pathway is also activated, promoting cell survival and proliferation. This helps restore tissues during inflammation resolution [25, 26]. However, following the binding and signal transduction of the IL-6/sIL-6R/gp130 complex, the phosphorylated STAT3 dimer translocates to the nucleus, promoting the transcription of pro-inflammatory cytokines like IL-1*β* and tumor necrosis factor-*α* (TNF-*α*), thereby intensifying the inflammatory response [27]. At the same time, activation of MAPK pathway leads to the production of pro-inflammatory cytokines and chemokines [28]. The IL-6/sIL-6R/gp130 complex also activates the NF-*κ*B pathway. Activated NF-*κ*B then translocates to the nucleus and forms dimers, promoting the transcription of pro-inflammatory genes [29–31]. Additionally, Sylvia Hein et al. [32] proposed in 2017 that IL-6 cluster signaling is the third mode of IL-6 signaling. Their research indicate that DCs are able to trans-present IL-6 through a complex containing DC-expressed IL-6R*α* bound to IL-6 that can interact with gp130 expressed on T cells, leading to the targeted activation of STAT3 in antigen specific T cells, mediating pathogenic T cell differentiation. Three IL-6 signaling pathways and downstream signal transduction in the cells can be seen in Figure 1.

IL-6 is a multifunctional cytokine that is produced by macrophages, dendritic cells, neutrophils, B cells, and some CD4+T cells. It is secreted by endothelial cells, fibroblasts, and epithelial cells [33]. IL-6 regulates the growth and differentiation of various tissues and promotes the activation of acute-phase proteins, including C-reactive protein (CRP) and fibrinogen, thereby inducing systemic inflammation [34, 35]. At the same time, many key factors in autoimmune diseases and cancer also play a role mainly through IL-6 signaling transduction factors and the STAT3 pathway [36]. Previous studies have shown that IL-6 is overexpressed in cardiovascular disease, osteoporosis, arthritis, type 2 diabetes, kidney disease, hepatitis, schizophrenia, preeclampsia, tumors, periodontal disease, and other diseases [37–42]. After central nervous system damage, such as traumatic brain injury, the expression of IL-6 in the cerebrospinal fluid increases, which can trigger the secretion of nerve growth factors in astrocytes [43]. Many studies have shown that IL-6 is closely related to the occurrence and development of OD in various diseases; however, its specific mechanism of action remains unclear. IL-6, as an endogenous substance, has been shown to regulate the activity of neurons and glial cells, and can be regulated by TNF-*α* or directly inhibit olfactory function by activating the apoptotic pathway through neurotransmitters [44]. The expression level of IL-6 in the olfactory system is closely related to olfactory function. In patients with OD, the expression levels of IL-6 are often increased, indicating that IL-6 may be involved in the pathological and physiological processes of OD. Neuroimmune inflammatory responses are key factors in the onset and progression of OD. As one of the central mediators of immune-inflammatory responses, IL-6 is closely associated with the pathogenesis of OD. A study by Ullah MN et al. highlighted that the olfactory epithelium not only serves a sensory function but also plays a role in immune defense. Chronic inflammation of the nasal mucosa is strongly linked to OD, and elevated IL-6 levels can impair the barrier function of nasal epithelial cells, triggering neuroinflammation and leading to olfactory impairment. Furthermore, through a positive feedback mechanism, increased IL-6 levels exacerbate OD, a finding consistent with the research of Song et al. [45, 46]. Additionally, a study by Leon M et al. demonstrated that elevated IL-6 can further promote the release of other inflammatory mediators, such as IL-1*β* and TNF-*α*, through a positive feedback loop, contributing to the deterioration of olfactory function. The release of these inflammatory factors, in turn, enhances IL-6 production, further exacerbating the inflammatory response [47]. Therefore, an in-depth study of the relationship between IL-6 and OD not only helps to reveal the pathogenesis of OD but may also provide new ideas and methods for the treatment of OD.

## 3. IL-6 and Disease-Related OD

### 3.1. IL-6 and COVID-19 Disease 2019-Related OD

COVID-19 disease 2019 (COVID-19) is a respiratory infectious disease caused by severe acute respiratory syndrome coronavirus type 2 (SARSCOVID-19). The main symptoms include dry cough, fever, fatigue, myalgia, arthralgia, headache, sore throat, nasal congestion, diarrhea, and dysosmia. Among them, OD is one of the most important symptoms of COVID-19, and 33%–80% of COVID-19 patients have dysosmia and taste disorders. Some studies suggest that OD is the earliest or even the only manifestation of COVID-19 [48, 49]. Yang et al. proposed that the role of IL-6 in the disease is of significant value, suggesting that peripheral blood IL-6 levels can serve as an independent factor in predicting the progression of COVID-19 [50, 51]. Similarly, Jose et al. [52] reported that IL-6 is associated with rapid disease progression and a high incidence of complications in COVID-19. Cazzolla et al. [11] suggested that an increase in IL-6 expression levels in patients with COVID-19 and OD is positively correlated with OD severity. Patients with higher IL-6 levels have higher scores for olfactory and taste dysfunction, and those with both symptoms have higher IL-6 levels.

Meanwhile, some scholars, such as Vaira et al. [53] have shown that IL-6 expression in COVID-19 is not significantly associated with OD. Conversely, some scholars, such as Yağmur et al. have demonstrated that IL-6 expression is lower in COVID-19 patients with OD than in those without, which is consistent with the findings of Dets et al. [54, 55], this phenomenon may be closely associated with the number of participants included, disease severity, or the sampling method and quality. The exact pathophysiology of taste and OD after COVID-19 infection is not yet clear, and hypotheses can only be formulated based on research on other coronaviruses. Currently, several mechanisms have been proposed: (a) central involvement is related to the ability of human coronaviruses to invade the olfactory bulb (OB) and thus spread to the central nervous system, (b) central involvement is related to the ability of viruses to enter the microcirculation of the brain and damage the brain, and (c) nasal epithelium involvement directly damages the peripheral olfactory receptor neurons (ORNs)[56]. However, the specific mechanism by which IL-6 acts in COVID-19-related OD needs to be further explored. Of course, the research of Zhang [57] and Mao et al. [58] found that IL-6 expression would also increase in influenza and viral pneumonia, and the research of Zhang J et al. showed that IL-6 can be an important factor to predict the severity of influenza related pneumonia, but whether IL-6 contributes to OD in these diseases has yet to be investigated.

### 3.2. IL-6 and Primary Sjögren's Syndrome (pSS) Related OD

pSS is a chronic systemic autoimmune rheumatic disease characterized by the infiltration of lymphatic plasma cells in the salivary and lacrimal glands. pSS is the most common connective tissue disease after rheumatoid arthritis, affecting 0.3%–3% of the population [59]. Activation of interferon regulatory factor (IRF) and nuclear factor *κ* light chain-enhancing factor-activated B-cell (nuclear factor kappa-B, NF-*κ*B) pathways increases the production of inflammatory cytokines, including IL-6, TNF-*α*, IL-1, and type I IFN [60]. Benchabane et al. reported a correlation between IL-6 and nitric oxide (NO) levels, suggesting that IL-6 participates in the pathological and physiological processes of pSS through the action of NO. Therefore, detecting IL-6 and NO in the serum and saliva of patients with suspected pSS can improve clinical diagnosis and prognosis [61]. Kawanami et al. [62] found that high concentrations of IL-6 in the supernatant of salivary gland epithelial cells (SGE) from patients with pSS may indicate the persistence of chronic inflammation, suggesting that IL-6 plays an important role in maintaining chronic inflammation in pSS. Xu et al. [12] demonstrated that both innate and adaptive immunity respond in pSS patients, leading to elevated levels of pro-inflammatory cytokines IL-6, IL-1, and TNF-*α*. These can lead to OD, and olfactory function is negatively correlated with the European League Against Rheumatology Sjögren's Syndrome Patient Reported Index (ESSPRI). This result also suggests that OD caused by an increase in inflammatory factors, such as IL-6 may be a manifestation of pSS activity; however, the specific mechanism has not yet been clarified. This study linked the OD of IL-6 and pSS and proposed a relationship with pSS activity. However, the specific regulatory mechanism requires further investigation.

### 3.3. IL-6 and Chronic Sinusitis-Related OD

Chronic rhinosinusitis (CRS) is one of the most common inflammatory diseases affecting the nose. There are two types of CRS: chronic rhinosinusitis without nasal polyps (CRSsNP) and chronic rhinosinusitis with nasal polyps (CRSwNP). CRSwNP is a typical Th2 type immune inflammatory response, accompanied by high eosinophil infiltration. Compared with patients with CRSsNP, those with CRSwNP exhibit more evident drug resistance during treatment, and 30%–80% of patients with CRS have olfactory disorders [63, 64]. The incidence of olfactory disorders in patients with CRSwNP is higher, which is the main reason for the decline in the quality of life in these patients [65–67]. In a study of the olfactory function of 59 patients with CRS, Henkin et al. [68] found that patients with OD had higher levels of IL-6 in their blood, urine, and nasal mucus. Wu et al. [13] collected olfactory cleft and middle nasal mucus samples from 31 patients with CRSsNP, 36 patients with CRSwNP, and 12 healthy controls. The results showed that patients with CRSwNP had significantly higher IL-6 levels and lower objective olfactory function than the other two groups. In the study by Soler et al. IL-6 levels in the olfactory cleft (OC) mucus of patients with CRSwNP were found to be negatively correlated with odor identification scores in the TDI test, indicating that higher IL-6 levels were associated with poorer olfactory function. This finding is consistent with the results reported by Wu et al. [69]. Although CRS is closely related to OD, the specific regulatory mechanism remains unclear. Most previous studies have proposed that olfactory decline and loss in CRS are the result of the combined effect of conductive and sensorineural OD. Currently, an increasing number of studies are being conducted on IL-6 in patients with CRS with OD, and the regulatory methods will become clearer.

### 3.4. IL-6 and Neurodegenerative Disease-Related OD

#### 3.4.1. IL-6 and PD-Related OD

Patients with PD exhibit various non-motor symptoms, including OD and depression, which are very common at various stages of the disease and may even appear before the appearance of motor features [70–72]. The main cause of PD is due to the degeneration of nigral dopaminergic neurons and the presence of Lewy bodies (LB) and Lewy neurons, which are associated with the misfolding of *α*-synuclein (*α*-syn). PD is usually unilateral or asymmetrical, with typical motor symptoms including tremors, unstable postures, and muscle stiffness, which seriously threaten the patient's motor function. In addition to typical motor symptoms, patients with early PD also have non-motor symptoms, such as OD, cognitive decline, sleep disorders, and autonomic dysfunction [73–75]. Kohli et al. have shown that OD not only contributes to the early diagnosis of PD, but also serves as a predictive indicator for some psychiatric symptoms, such as depression. The dysfunction of pro-inflammatory cytokines such as, IL-6 can lead to olfactory loss [76–78]. Li et al. suggested that after inducing olfactory mucositis, the levels of IL-6 within the OB increase, leading to OD. The pro-inflammatory activity induced by IL-6 may play an important role in the development of PD precursors [79–81]. This shows that the alteration of the inflammatory factor IL-6 in patients with PD is related to their OD, and IL-6 has attracted attention in PD-associated OD, but has been less studied to date.

#### 3.4.2. IL-6 and Alzheimer's Disease (AD)-Related OD

AD is a neurodegenerative disorder. Soo et al. suggested that patients with more severe cognitive impairment have poorer olfactory function [82]. Activation of microglial cells (MG), characterized by elevated levels of inflammatory factors, is the core pathological mechanism of AD [83, 84]. Daulatzai et al. [85] suggested that the earliest pathological changes in AD include OD, entorhinal cortex atrophy, and hippocampal atrophy, and that OD is an equally important early correlate of AD pathogenesis. Garamszegi et al. [15] found that elevated IL-6 was observed in the OB of patients with AD, and that the presence of IL-6 and cyanobacterial toxin (*β*-Methylamino-L-alanine [BMAA]) in the olfactory microenvironment may exacerbate olfactory neuronal damage, leading to OD. Wang et al. [86] showed that “xiu san zhen” activated the olfactory pathway to improve the learning ability of AD mice and exerted an inhibitory effect on inflammatory factors, one of the mechanisms may be the inhibition of the release of inflammatory factors, such as IL-6 and IL-1*β*, to reduce the inflammatory response triggered by microglial activation. Rao et al. showed that the total peripheral blood expression of TNF-*α* and IL-6 was elevated in patients with AD [87, 88]. Notably, in a study by Soares et al. [89] IL-6 levels were found to be significantly lower in the peripheral blood of patients with AD. However, Li et al. [90] categorized patients with AD into groups with and without OD and found no statistically significant difference in IL-1*β*, IL-6, and TNF-*α* between the two groups. This may be closely related to the number of participants included, disease severity, or the sampling method and quality.

### 3.5. IL-6 and OD Related to Traumatic Brain Injury

Head trauma, a disease caused by an outside force, can cause OD through hyperextension, twisting, and tearing of the olfactory nerves, as well as contusions to the OB and orbitofrontal region of the brain. Although the olfactory system undergoes remarkable neuroregeneration and recovery after injury, olfactory function cannot be restored in many severe head injuries [91]. Recent research has shown that the incidence of OD after brain injury is 11%–22% [92]. Kobayashi et al. [14] found that IL-6 promoted microglial activation and infiltration, and aggravated tissue damage during inflammation, whereas anti-mouse IL-6R antibody inhibited local inflammatory cell infiltration and promoted functional recovery of the olfactory system in a dose-dependent manner. Thus, blocking IL-6R may be a novel therapeutic strategy for the treatment of OD after craniocerebral injury. Macrophages/microglia are the main inflammatory cells in CNS injury, and CD68 is prominently expressed on macrophages and activated microglia, both of which are found in damaged tissues. Therefore, CD68 can be used as a good indicator to monitor the extent of the inflammatory process [93]. Kobayashi et al. [94] showed that anti IL-6R antibodies can reduce the number of CD68-positive cells in damaged OBs, indicating that blocking IL-6R and steroids can inhibit local inflammation. However, Swartz et al. studied the IL-6 gene knockout and showed that completely blocking the IL-6 signaling pathway could inhibit axonal regeneration or lead to glial cell atrophy and tissue necrosis, which is not conducive to the functional recovery of the damaged CNS [95–97]. This is consistent with the protective effect of IL-6 on nerve regeneration in some neurological injuries, central ischemic injuries, and neuroinflammation mentioned earlier.

## 4. IL-6 Regulation in the Olfactory Function

### 4.1. Expression and Regulation of IL-6 in the Olfactory System

IL-6 is a crucial inflammatory factor that plays a significant role in the physiological and pathological processes of olfactory neurons. In a clinical sample study, Henkin et al. [68] revealed a significant increase in IL-6 levels in the peripheral blood, saliva, and nasal mucus of patients with olfactory impairment. In animal experiments, Zhiming et al. confirmed that the levels of IL-6 in the serum and nasal mucosa of OD model rats were significantly increased. The ongoing systemic inflammatory response can also affect the recovery of the nasal mucosal structure, leading to increased damage [98]. By constructing an animal model, researchers found that the expression level of IL-6 was significantly elevated during the development of OD. They detected a decrease in neuroinflammatory indices in the OB after inhibiting the expression of IL-6 using small interfering RNA of IL-6R in in vitro experiments, which indicated that neuroinflammation in the olfactory bulb was an important cause of OD development [46]. Studies on olfaction found that IL-6 affects the nuclear factor NF-*κ*B and the adenosine triphosphate-ubiquitin-dependent protein hydrolysis pathway to activate tumor necrosis factor, which activates the apoptotic pathways of olfactory functions [99, 100]. Most current research has focused on the correlation between OD and inflammatory indicators, such as IL-6; however, the specific pathways and mechanisms of action remain unclear. This suggests that changes in the levels of inflammatory factors, such as IL-6, may further exacerbate OD development. Chronic exposure to irritants, such as the herbicide 2,6-dichlorobenzonitrile (DCBN) can cause chronic elevation of nasal IL-6 levels, which results in damage to nasal olfactory mucosal supporting and basal cells and the inability of residual and repopulated basal cells to differentiate into ORNs, leading to a reduction in the number of olfactory neurons and impaired function; a mechanism that explains OD manifestations in patients with elevated IL-6 levels is clearer [101].

Based on these findings, is the symptoms of OD are expected to improve by inhibiting the expression of IL-6 or blocking its signaling pathway. However, these therapeutic strategies present several challenges. A deeper understanding of the specific mechanism of IL-6 action in the olfactory system could allow for more precise regulation of its expression and provide better treatments for patients with OD.

### 4.2. IL-6 and Olfactory Neuron Injury

The olfactory epithelium is located bilaterally in the upper nasal cavity, and contains heterogeneous cell populations [102]. Mature olfactory neurons are bipolar neurons that detect odors on the ciliated dendritic tips facing the nasal cavity. The olfactory-driven activity is then transmitted by axons that form small bundles of the olfactory nerve and pass through the ethmoid foramen of the ethmoid plate. Mature ORNs in the olfactory epithelium project to the OB and communicate with higher-level areas of the brain, including the cortex, amygdala, and hippocampus [103, 104]. IL-6 is widely expressed in various neurons and regulated by various factors. When olfactory neurons are stimulated by external odors, they trigger a series of signaling pathways, including the expression and release of IL-6. The changes in this expression pattern may be related to the response and adaptability of olfactory neurons to different odors, and the expression of IL-6 is regulated by various factors, such as TNF-*α* and IL-1 [105]. The olfactory marker protein (OMP) and growth-associated protein 43 (GAP43) serve as markers for mature and early maturing ORNs, respectively [106–108]. Zhang et al. [109] indicated that under anesthesia and surgical induction conditions, the number of presynaptic markers and postsynaptic density (excitatory postsynaptic markers) decreases, which in turn causes an elevation in IL-6 levels in the blood and olfactory epithelium of mice, leading to a reduction in the number of olfactory sensory neurons (OSNs), resulting in OD and cognitive impairment.

Pro-inflammatory cytokines are generally considered detrimental to neuronal regeneration; however, the effect of IL-6 on olfactory neurons is not entirely negative. Appropriate levels of IL-6 can promote the regeneration and repair of olfactory neurons. Lane et al. [110] demonstrated a more complex role of inflammatory factors leading to OSN injury, with the expression of macrophages, TNF-*α*, IL-1*β*, and IL-6 increasing within a few days after methimazole or methyl bromide application, and inhibition of these transient inflammatory responses instead impairing OSN regeneration. These studies support the need for inflammatory factors to be involved in OSN repair; however, tightly controlling their expression and the timing of expression is crucial. Similar studies have shown that IL-6 has a protective effect on nerve regeneration in some neurological injuries, central ischemic injury, and neuroinflammation [111–113]. Therefore, maintaining the beneficial effects of IL-6 on olfactory neurons while suppressing its harmful effects is an important direction for future research.

In summary, the effects of IL-6 on olfactory neurons are dual, which may lead to damage of olfactory neurons by inducing apoptosis and necrosis, and promoting the regeneration and repair of olfactory neurons at a certain concentration or effect time. Therefore, an in-depth study on the specific mechanism of action of IL-6 in olfactory neurons is important for the development of effective therapeutic approaches against OD.

## 5. Summary and Prospect

Although there has been increasing clinical attention to OD, prevention and treatment of OD remain challenging. At present, the treatment plans for OD mainly include the following: (i) treatment of the etiology of the disease if there is a clear primary cause; (ii) pharmacological treatment with glucocorticoids, vitamin A, ginkgo biloba, and other agents, to improve the local inflammation or nutritional support of the nerves [114–116]; (iii) surgical treatment of deviated septum and nasal polyps and similar interventions to improve the structure of the nasal cavity and ensure that the olfactory molecules can effectively contact the olfactory zone effector cells or neurons; (iv) olfactory training treatment; and (v) other treatments. In addition to the medicinal and surgical treatments mentioned above, some physical therapies, such as olfactory training, are also widely used in the treatment of OD. Olfactory training is a cognitive–behavioral intervention to enhance olfactory ability through repeated sniffing and inhalation of various olfactory agents and is supported by level 1A evidence. Since Hummel et al. first pointed out in 2009 that patients with OD can enhance their olfactory function through olfactory training, many studies have confirmed that olfactory training can be useful for the treatment of OD caused by viral infection, trauma, and neurological injury, helping to restore the function of the olfactory nerve, improve the olfactory recognition ability of patients with OD, and improve their quality of life [117–119]. However, these methods have limitations.

There are currently various methods for olfactory detection, such as the University of Pennsylvania Smell Identification Test (UPSIT), Sniffin 'Sticks Test, Odor Identification Task (OIT), and so on. Among them, UPSIT is currently the most widely used for olfactory recognition and detection. It consists of 40 items, each corresponding to a common odor [120, 121]. Each correct recognition earns 1 point, with a maximum score of 40 points. A score below 30 indicates that the patient has olfactory impairment. UPSIT is widely used in research, clinical trials, and medical diagnosis due to its easily recognizable odor and high sensitivity. It cannot be denied that UPSIT has its detection advantages, but there are also shortcomings. For example, in poverty-stricken or medically underdeveloped areas, there are still certain difficulties in operating UPSIT, and this test also requires professional personnel to guide the operation, which increases manpower to a certain extent. The detection of IL-6 only requires the extraction of 3–5 ml of blood sample, which can be completed in the laboratory of a regular hospital without the need for separate professional personnel. Therefore, theoretically, the detection completion rate of IL-6 is higher than that of UPSIT. At present, IL-6 is associated with OD in many diseases. When patients' economic conditions permit, psychophysical testing combined with IL-6 can be used for detection, we can not only measure changes in olfactory ability, but also understand the degree of OD and potential recovery ability through monitoring IL-6 levels. Therefore, IL-6 is not only a diagnostic tool, but also provides clues about the mechanisms of OD. IL-6 can serve as both a diagnostic marker and a therapeutic marker, and is a simple and reproducible biomarker with high clinical potential, especially in early screening, diagnosis, and monitoring of disease progression.

With the gradual increase in studies on the correlation between IL-6 and OD, it has been observed that IL-6 is highly expressed, has a positive correlation with OD in most OD-related diseases, and plays an important regulatory role in the maintenance of the structure and function of the olfactory system. Based on the above, IL-6 is not only a marker of OD, but also may be a potential target in OD diagnosis. Some therapeutic approaches targeting IL-6 are available, such as tocilizumab (TCZ), the first IL-6-targeted biologic approved for the treatment of various inflammatory diseases (including rheumatoid arthritis and systemic arthritis). TCZ inhibits IL-6 signaling through targeted binding to IL-6R*α* [122]. Currently, there are four potential extracellular targets: the IL-6 signaling pathway, IL-6R, gp130, and IL-6/sIL-6R complexes [123]. However, the application of this method to detect whether OD has recovered or can be improved during the course of treatment has not yet been reported. No drugs have been used to treat OD-related diseases, and further studies are needed to determine whether drugs that inhibit IL-6 can be used for the clinical treatment of OD. In a study of allergic rhinitis (AR) with OD, our team found that the inhibition of IL-6 in an OD model may improve OD [46]. IL-6 can affect nerve regeneration in olfaction, therefore, whether IL-6 can be combined with stem cells to treat OD, as well as gene editing technology and cell therapy, are also directions for future research.

At the same time, we also acknowledge that there are still many shortcomings in the current research status of IL-6, such as the lack of a unified cutoff value, and the lack of differentiation in expression based on race and gender. As age increases, changes in olfactory related anatomical structures and functional degradation of the olfactory nerve can also cause olfactory decline [124]. It is currently unclear whether these olfactory disorders are accompanied by changes in IL-6, so it is more worthwhile for us to further establish a prospective cohort study for further exploration. Of course, it cannot be said that a single marker can determine all aspects of diagnosis. In the future, combining IL-6 with UPSIT testing is likely to demonstrate greater advantages. However, the economic implications of such combined testing must also be considered, and thus, the balance must be sought in clinical practice. We expect that with the deepening of research and development of technology, we will find more therapeutic approaches targeting IL-6, which will bring about better therapeutic effects and quality of life for OD patients.

## Figures and Tables

**Table 1:** Expression of IL-6 in diseases related to olfactory.

Disease	Assessment of olfactory disorders		Measurement of IL-6		The relationship between the expression of IL-6 and olfactory disorders in diseases	Possible mechanisms	Citation information
	Assessment scale	Physical assessment methods	Detection location	Detection method			
COVID-19	Taste and smell Questionnaire section (CSQ) of the US NHANES 2011−2014 protocol (CDC 2013 b)	Sinonasal outcome test 22 (SNOT-22)	Peripheral blood	Chemiluminescence assay	High levels of IL-6 associated with olfactory and taste deficits in COVID-19 patients	Viral damage to neuronal cells or ischemic damage to the central nervous system	[11]
Primary Sjögren syndrome	Visual analog scale (VAS), olfactory function assessment by computerized testing (OLFACT)	Olfactory threshold (THR),identification (ID) and memory (ME)	Peripheral blood	Enzyme-linked immunosorbent assay	IL-6 is positively correlated with the severity of olfactory dysfunction	IL-6 proliferation reduces neurogenesis in the hippocampus, amygdala, and affects olfactory neuronal effect	[12]
Chronic rhinosinusitis	None	Smell Identification Test(SIT)	Nasal mucus	Flow cytometry	Higher IL-6 levels in mucus of CRS with OD patients compared to healthy controls	Elevated IL-6 is associated with reduced neurogenesis	[13]
Traumatic brain injury	Mouse behavioral tests	Olfactory avoidance behavioral test and Electric field potential record	OB	X-gal staining and immunohistochemical assessments	Anti-mouse IL-6R antibody inhibited local inflammatory cell infiltration and promoted functional recovery of the olfactory system in a dose-dependent manner	IL-6 promoted microglial activation, infiltration, and aggravated tissue damage during inflammation	[14]
Alzheimer's disease	Autopsy	Autopsy	OB	qPCR	IL-6 gene expression is upregulated	Chronic exposure to toxin invasion of olfactory tissues may increase gene expression profiles, leading to neuroinflammation and olfactory dysfunction in susceptible individuals	[15]

Abbreviation: OB, olfactory bulb.

## Data Availability

No datasets were generated or analyzed during the current study.

## References

[1] Schäfer L., Schriever V. A., Croy I. (2021). Human Olfactory Dysfunction: Causes and Consequences. *Cell and Tissue Research*.

[2] Ren C., Mou Y. K., Song X. Y. (2023). P2X7 Receptor of Microglia in Olfactory Bulb Mediates the Pathogenesis of Olfactory Dysfunction in a Mouse Model of Allergic Rhinitis. *FASEB Journal*.

[3] Consensus on the Diagnosis and Treatment of Olfactory Disorders (2017). Editorial Committee of the Chinese Journal of Otolaryngology-Head and Neck Surgery, Rhinology Group, Otolaryngology-Head and Neck Surgery Branch of the Chinese Medical Association. *Chinese Journal of Otolaryngology-Head and Neck Surgery*.

[4] Wei Y. X., Han D. M. (1998). The Current Research Status of Olfactory Disorders. *Chinese Journal of Otorhinolaryngology Head and Neck Surgery*.

[5] Rombaux P., Mouraux A., Bertrand B., Nicolas G., Duprez T., Hummel T. (2006). Olfactory Function and Olfactory Bulb Volume in Patients with Postinfectious Olfactory Loss. *Laryngoscope*.

[6] Ross G. W., Petrovitch H., Abbott R. D. (2008). Association of Olfactory Dysfunction with Risk for Future Parkinson’s Disease. *Annals of Neurology*.

[7] Damm M., Schmitl L., Müller C. A., Welge-Lüssen A., Hummel T. (2019). Diagnostik Und Therapie Von Riechstörungen [Diagnostics and Treatment of Olfactory Dysfunction]. *HNO*.

[8] Förster G., Damm M., Gudziol H. (2004). Epidemiologie, Pathophysiologische Klassifikation, Diagnose Und Therapie [Olfactory Dysfunction. Epidemiology, Pathophsiological Classification, Diagnosis and Therapy]. *HNO*.

[9] Su B., Bleier B., Wei Y., Wu D. (2021). Clinical Implications of Psychophysical Olfactory Testing: Assessment, Diagnosis, and Treatment Outcome. *Frontiers in Neuroscience*.

[10] Miwa T., Ikeda K., Ishibashi T. (2019). Clinical Practice Guidelines for the Management of Olfactory Dysfunction—Secondary Publication. *Auris Nasus Larynx*.

[11] Cazzolla A. P., Lovero R., Lo Muzio L. (2020). Taste and Smell Disorders in COVID-19 Patients: Role of Interleukin-6. *ACS Chemical Neuroscience*.

[12] Xu X., Geng L., Chen C. (2021). Olfactory Impairment in Patients with Primary Sjogren’s Syndrome and Its Correlation with Organ Involvement and Immunological Abnormalities. *Arthritis Research & Therapy*.

[13] Wu J., Chandra R. K., Li P., Hull B. P., Turner J. H. (2018). Olfactory and Middle Meatal Cytokine Levels Correlate with Olfactory Function in Chronic Rhinosinusitis. *Laryngoscope*.

[14] Kobayashi M., Tamari K., Miyamura T., Takeuchi K. (2013). Blockade of Interleukin-6 Receptor Suppresses Inflammatory Reaction and Facilitates Functional Recovery following Olfactory System Injury. *Neuroscience Research*.

[15] Garamszegi S. P., Banack S. A., Duque L. L. (2023). Detection of *β*-N-Methylamino-l-Alanine in Postmortem Olfactory Bulbs of Alzheimer’s Disease Patients Using UHPLC-MS/MS:An Autopsy Case-Series Study. *Toxicology Reports*.

[16] Murakami M., Kamimura D., Hirano T. (2019). Pleiotropy and Specificity: Insights from the Interleukin 6 Family of Cytokines. *Immunity*.

[17] Masjedi A., Hashemi V., Hojjat-Farsangi M. (2018). The Significant Role of Interleukin-6 and Its Signaling Pathway in the Immunopathogenesis and Treatment of Breast Cancer. *Biomedicine & Pharmacotherapy*.

[18] Akbari M., Hassan-Zadeh V. (2018). IL-6 Signalling Pathways and the Development of type 2 diabetes. *Inflammopharmacology*.

[19] Pandolfi R., Barreira B., Moreno E. (2017). Role of Acid Sphingomyelinase and IL-6 as Mediators of Endotoxin-Induced Pulmonary Vascular Dysfunction. *Thorax*.

[20] Rose-John S. (2018). Interleukin-6 Family Cytokines. *Cold Spring Harbor Perspectives in Biology*.

[21] Scheller J., Chalaris A., Garbers C., Rose-John S. (2011). ADAM17: A Molecular Switch to Control Inflammation and Tissue Regeneration. *Trends in Immunology*.

[22] Xu J., Peng W. R., Zhang D. (2024). Marine Sponge-Derived Alkaloid Ameliorates DSS-Induced IBD via Inhibiting IL-6 Expression through Modulating JAK2-STAT3-SOCS3 Pathway. *International Immunopharmacology*.

[23] Garbers C., Aparicio-Siegmund S., Rose-John S. (2015). The IL-6/gp130/STAT3 Signaling Axis: Recent Advances towards Specific Inhibition. *Current Opinion in Immunology*.

[24] Swaroop A. K., Negi P., Kar A. (2024). Navigating IL-6: From Molecular Mechanisms to Therapeutic Breakthroughs. *Cytokine & Growth Factor Reviews*.

[25] Shan C., Zhang C., Zhang C. (2024). The Role of IL-6 in Neurodegenerative Disorders. *Neurochemical Research*.

[26] Schaper F., Rose-John S. (2015). Interleukin-6: Biology, Signaling and Strategies of Blockade. *Cytokine & Growth Factor Reviews*.

[27] Xu J., Lin H., Wu G., Zhu M., Li M. (2021). IL-6/STAT3 Is a Promising Therapeutic Target for Hepatocellular Carcinoma. *Frontiers in Oncology*.

[28] Saad M. I., Alhayyani S., McLeod L. (2019). ADAM17 Selectively Activates the IL-6 Trans-Signaling/ERK MAPK Axis in KRAS-Addicted Lung Cancer. *EMBO Molecular Medicine*.

[29] Garbers C., Heink S., Korn T., Rose-John S. (2018). Interleukin-6: Designing Specific Therapeutics for a Complex Cytokine. *Nature Reviews Drug Discovery*.

[30] Kang S., Narazaki M., Metwally H., Kishimoto T. (2020). Historical Overview of the Interleukin-6 Family Cytokine. *Journal of Experimental Medicine*.

[31] Osman E. E. A., Neamati N. (2024). Ironing Out the Mechanism of gp130 Signaling. *Pharmacological Reviews*.

[32] Heink S., Yogev N., Garbers C. (2017). Trans-Presentation of IL-6 by Dendritic Cells Is Required for the Priming of Pathogenic TH17 Cells. *Nature Immunology*.

[33] Gubernatorova E. O., Gorshkova E. A., Namakanova O. A. (2018). Non-Redundant Functions of IL-6 Produced by Macrophages and Dendritic Cells in Allergic Airway Inflammation. *Frontiers in Immunology*.

[34] Yudkin J. S., Stehouwer C. D., Emeis J. J., Coppack S. W. (1999). C-Reactive Protein in Healthy Subjects: Associations with Obesity, Insulin Resistance, and Endothelial Dysfunction: A Potential Role for Cytokines Originating from Adipose Tissue?. *Arteriosclerosis Thrombosis and Vascular Biology*.

[35] Koukkunen H., Penttilä K., Kemppainen A. (2009). C-Reactive Protein, Fibrinogen, Interleukin-6 and Tumour Necrosis Factor-Alpha in the Prognostic Classification of Unstable Angina Pectoris. *Annals of Medicine*.

[36] Hirano T. (2021). IL-6 in Inflammation, Autoimmunity and Cancer. *International Immunology*.

[37] Kiecolt-Glaser J. K., Preacher K. J., MacCallum R. C., Atkinson C., Malarkey W. B., Glaser R. (2003). Chronic Stress and Age-Related Increases in the Proinflammatory Cytokine IL-6. *Proceedings of the National Academy of Sciences of the United States of America*.

[38] Pickup J. C., Chusney G. D., Thomas S. M., Burt D. (2000). Plasma Interleukin-6, Tumour Necrosis Factor Alpha and Blood Cytokine Production in type 2 diabetes. *Life Science Part 1 Physiology & Pharmacology*.

[39] Sheron N., Bird G., Goka J., Alexander G., Williams R. (1991). Elevated Plasma Interleukin-6 and Increased Severity and Mortality in Alcoholic Hepatitis. *Clinical and Experimental Immunology*.

[40] Maes M., Meltzer H. Y., Bosmans E. (1994). Immune-Inflammatory Markers in Schizophrenia: Comparison to Normal Controls and Effects of Clozapine. *Acta Psychiatrica Scandinavica*.

[41] Taniguchi K., Karin M. (2014). IL-6 and Related Cytokines as the Critical Lynchpins between Inflammation and Cancer. *Seminars in Immunology*.

[42] Lai H. S., Lin W. H., Lai S. L. (2013). Interleukin-6 Mediates Angiotensinogen Gene Expression during Liver Regeneration. *PLoS One*.

[43] Kossmann T., Hans V., Imhof H. G., Trentz O., Morganti-Kossmann M. C. (1996). Interleukin-6 Released in Human Cerebrospinal Fluid following Traumatic Brain Injury May Trigger Nerve Growth Factor Production in Astrocytes. *Brain Research*.

[44] Kummer K. K., Zeidler M., Kalpachidou T., Kress M. (2021). Role of IL-6 in the Regulation of Neuronal Development, Survival and Function. *Cytokine*.

[45] Ullah M. N., Rowan N. R., Lane A. P. (2024). Neuroimmune Interactions in the Olfactory Epithelium: Maintaining a Sensory Organ at an Immune Barrier Interface. *Trends in Immunology*.

[46] Song X. Y., Sun Q., Wei S. Z. (2024). IL-6 Mediates Olfactory Dysfunction in a Mouse Model of Allergic Rhinitis. *Brain Research*.

[47] Leon M., Troscianko E. T., Woo C. C. (2018). Inflammation and Olfactory Loss Are Associated with at least 139 medical Conditions. *Frontiers in Molecular Neuroscience*.

[48] Wang D., Hu B., Hu C. (2020). Clinical Characteristics of 138 Hospitalized Patients With 2019 Novel Coronavirus-Infected Pneumonia in Wuhan, China. *JAMA*.

[49] Mao L., Jin H., Wang M. (2020). Neurologic Manifestations of Hospitalized Patients With Coronavirus Disease 2019 in Wuhan, China. *JAMA Neurology*.

[50] Yang P. H., Ding Y. B., Xu Z. (2020). Increased Circulating Level of Interleukin-6 and CD8+ T Cell Exhaustion Are Associated with Progression of COVID-19. *Infectious Diseases of Poverty*.

[51] Liu F., Li L., Xu M. (2020). Prognostic Value of Interleukin-6, C-Reactive Protein, and Procalcitonin in Patients with COVID-19. *Journal of Clinical Virology*.

[52] Jose R. J., Manuel A. (2020). COVID-19 Cytokine Storm: The Interplay between Inflammation and Coagulation. *Lancet Respiratory Medicine*.

[53] Vaira L. A., De Vito A., Deiana G. (2022). Correlations between IL-6 Serum Level and Olfactory Dysfunction Severity in COVID-19 Patients: A Preliminary Study. *European Archives of Oto-Rhino-Laryngology*.

[54] Yağmur A. R., Akbal Çufalı Ş., Aypak A., Köksal M., Güneş Y. C., Özcan K. M. (2022). Correlation of Olfactory Dysfunction with Lung Involvement and Severity of COVID-19. *Irish Journal of Medical Sciences*.

[55] Sanli D. E. T., Altundag A., Kandemirli S. G. (2021). Relationship between Disease Severity and Serum IL-6 Levels in COVID-19 Anosmia. *American Journal of Otolaryngology*.

[56] Baig A. M., Khaleeq A., Ali U., Syeda H. (2020). Evidence of the COVID-19 Virus Targeting the CNS: Tissue Distribution, Host-Virus Interaction, and Proposed Neurotropic Mechanisms. *ACS Chemical Neuroscience*.

[57] Zhang J., Wang J., Gong Y., Gu Y., Xiang Q., Tang L. L. (2022). Interleukin-6 and Granulocyte Colony-Stimulating Factor as Predictors of the Prognosis of Influenza-Associated Pneumonia. *BMC Infectious Diseases*.

[58] Mao Y., Bajinka O., Tang Z., Qiu X., Tan Y. (2022). Lung-Brain Axis: Metabolomics and Pathological Changes in Lungs and Brain of Respiratory Syncytial Virus-Infected Mice. *Journal of Medical Virology*.

[59] Parisis D., Chivasso C., Perret J., Soyfoo M. S., Delporte C. (2020). Current State of Knowledge on Primary Sjögren’s Syndrome, an Autoimmune Exocrinopathy. *Journal of Clinical Medicine*.

[60] Iwanaszko M., Kimmel M. (2015). NF-*κ*B and IRF Pathways: Cross-Regulation on Target Genes Promoter Level. *BMC Genomics*.

[61] Benchabane S., Boudjelida A., Toumi R., Belguendouz H., Youinou P., Touil-Boukoffa C. (2016). A Case for IL-6, IL-17A, and Nitric Oxide in the Pathophysiology of Sjögren’s Syndrome. *International Journal of Immunopathology and Pharmacology*.

[62] Kawanami T., Sawaki T., Sakai T. (2012). Skewed Production of IL-6 and TGF*β* by Cultured Salivary Gland Epithelial Cells from Patients with Sjögren’s Syndrome. *PLoS One*.

[63] Katotomichelakis M., Simopoulos E., Zhang N. (2013). Olfactory Dysfunction and Asthma as Risk Factors for Poor Quality of Life in Upper Airway Diseases. *American Journal of Rhinology & Allergy*.

[64] Simopoulos E., Katotomichelakis M., Gouveris H., Tripsianis G., Livaditis M., Danielides V. (2012). Olfaction-Associated Quality of Life in Chronic Rhinosinusitis: Adaptation and Validation of an Olfaction-Specific Questionnaire. *Laryngoscope*.

[65] Yan X., Whitcroft K. L., Hummel T. (2020). Olfaction: Sensitive Indicator of Inflammatory Burden in Chronic Rhinosinusitis. *Laryngoscope Investig Otolaryngol*.

[66] Thompson C. F., Price C. P., Huang J. H. (2016). A Pilot Study of Symptom Profiles from a Polyp Vs an Eosinophilic-Based Classification of Chronic Rhinosinusitis. *International Forum of Allergy & Rhinology*.

[67] Tsybikov N. N., Egorova E. V., Kuznik B. I., Fefelova E. V., Magen E. (2016). Neuron-Specific Enolase in Nasal Secretions as a Novel Biomarker of Olfactory Dysfunction in Chronic Rhinosinusitis. *American Journal of Rhinology & Allergy*.

[68] Henkin R. I., Schmidt L., Velicu I. (2013). Interleukin 6 in Hyposmia. JAMA Otolaryngol Head Neck Surg.

[69] Soler Z. M., Yoo F., Schlosser R. J. (2020). Correlation of Mucus Inflammatory Proteins and Olfaction in Chronic Rhinosinusitis. *International Forum of Allergy & Rhinology*.

[70] Siderowf A., Stern M. B. (2006). Preclinical Diagnosis of Parkinson’s Disease: Are We There yet?. *Current Neurology and Neuroscience Reports*.

[71] Schuurman A. G., van den Akker M., Ensinck K. T. J. L. (2002). Increased Risk of Parkinson’s Disease after Depression: A Retrospective Cohort Study. *Neurology*.

[72] Hawkes C. H. (2008). The Prodromal Phase of Sporadic Parkinson’s Disease: Does It Exist and if so How Long Is It?. *Movement Disorders*.

[73] Kalia L. V., Lang A. E. (2015). Parkinson’s Disease. *Lancet*.

[74] Berardelli A., Wenning G. K., Antonini A. (2013). EFNS/MDS-ES/ENS [corrected] Recommendations for the Diagnosis of Parkinson’s Disease. *European Journal of Neurology*.

[75] Doty R. L. (2012). Olfactory Dysfunction in Parkinson Disease. *Nature Reviews Neurology*.

[76] Kohli P., Soler Z. M., Nguyen S. A., Muus J. S., Schlosser R. J. (2016). The Association Between Olfaction and Depression: A Systematic Review. *Chemical Senses*.

[77] Bohnen N. I., Studenski S. A., Constantine G. M., Moore R. Y. (2008). Diagnostic Performance of Clinical Motor and Non-Motor Tests of Parkinson Disease: A Matched Case-Control Study. *European Journal of Neurology*.

[78] Tissingh G., Berendse H. W., Bergmans P. (2001). Loss of Olfaction in De Novo and Treated Parkinson’s Disease: Possible Implications for Early Diagnosis. *Movement Disorders*.

[79] Herbert R. P., Harris J., Chong K. P., Chapman J., West A. K., Chuah M. I. (2012). Cytokines and Olfactory Bulb Microglia in Response to Bacterial Challenge in the Compromised Primary Olfactory Pathway. *Journal of Neuroinflammation*.

[80] Bottigliengo D., Foco L., Seibler P., Klein C., König I. R., Del Greco M F. (2022). A Mendelian Randomization Study Investigating the Causal Role of Inflammation on Parkinson’s Disease. *Brain*.

[81] Li H., Qian J., Wang Y. (2024). Potential Convergence of Olfactory Dysfunction in Parkinson’s Disease and COVID-19: The Role of Neuroinflammation. *Ageing Research Reviews*.

[82] Yoo H. S., Jeon S., Chung S. J. (2018). Olfactory Dysfunction in Alzheimer’s Disease- and Lewy Body-Related Cognitive Impairment. *Alzheimers & Dementia*.

[83] Krstic D., Knuesel I. (2013). Deciphering the Mechanism Underlying Late-Onset Alzheimer Disease. *Nature Reviews Neurology*.

[84] Serrano-Pozo A., Muzikansky A., Gómez-Isla T. (2013). Differential Relationships of Reactive Astrocytes and Microglia to Fibrillar Amyloid Deposits in Alzheimer Disease. *Journal of Neuropathology & Experimental Neurology*.

[85] Daulatzai M. A. (2015). Olfactory Dysfunction: Its Early Temporal Relationship and Neural Correlates in the Pathogenesis of Alzheimer’s Disease. *Journal of Neural Transmission (Vienna)*.

[86] Wang Y., Liu Z. B., Niu W. M., Q. WANG (2018). Influence of “Xiusanzhen” Electroacupuncture on Learning Ability and IL1-*β*, IL-6 in Hippocamus for Mice with Alzheimer’s Disease. *Sichuan Traditional Chinese Medicine*.

[87] Rao J. S., Kellom M., Kim H. W., Rapoport S. I., Reese E. A. (2012). Neuroinflammation and Synaptic Loss. *Neurochemical Research*.

[88] Marksteiner J., Kemmler G., Weiss E. M. (2011). Five Out of 16 Plasma Signaling Proteins Are Enhanced in Plasma of Patients with Mild Cognitive Impairment and Alzheimer’s Disease. *Neurobiology of Aging*.

[89] Soares H. D., Potter W. Z., Pickering E. (2012). Biomarkers Consortium Alzheimer’s Disease Plasma Proteomics Project. Plasma bBiomarkers aAssociated with the aApolipoprotein E gGenotype and Alzheimer dDisease. *Archives of Neurology*.

[90] Zuo L. J., Guo P., Liu L. (2018). Analyses of Clinical Features and Investigations on Potential Mechanisms in Patients with Alzheimer’s Disease and Olfactory Dysfunction. *Journal of Alzheimers Disease*.

[91] Zasler N.D., Katz D. I., Zafonte R. D. (2007). Brain Injury Medicine: Principles and Practice.

[92] Sigurdardottir S., Jerstad T., Andelic N., Roe C., Schanke A. K. (2010). Olfactory Dysfunction, Gambling Task Performance and Intracranial Lesions after Traumatic Brain Injury. *Neuropsychology*.

[93] Lacroix S., Chang L., Rose-John S., Tuszynski M. H. (2002). Delivery of Hyper-Interleukin-6 to the Injured Spinal Cord Increases Neutrophil and Macrophage Infiltration and Inhibits Axonal Growth. *Journal of Comparative Neurology*.

[94] Kobayashi M., Costanzo R. M. (2009). Olfactory Nerve Recovery following Mild and Severe Injury and the Efficacy of Dexamethasone Treatment. *Chemical Senses*.

[95] Cafferty W. B., Gardiner N. J., Das P., Qiu J., McMahon S. B., Thompson S. W. (2004). Conditioning Injury-Induced Spinal Axon Regeneration Fails in Interleukin-6 Knock-Out Mice. *Journal of Neuroscience*.

[96] Swartz K. R., Liu F., Sewell D. (2001). Interleukin-6 Promotes Post-Traumatic Healing in the Central Nervous System. *Brain Research*.

[97] Okada S., Nakamura M., Katoh H. (2006). Conditional Ablation of Stat3 or Socs3 Discloses a Dual Role for Reactive Astrocytes after Spinal Cord Injury. *Nature Medicine*.

[98] Jiang Z. M., Wei X. T., Zhao L. N. (2021). [Effect of Different Concentrations of Moxa Smoke Exposure on Nasal Mucosal Injury and Expression of Serum IL-1, IL-6 and TNF-*α* in Rats]. *Zhongguo Zhen Jiu*.

[99] Blazka M. E., Germolec D. R., Simeonova P., Bruccoleri A., Pennypacker K. R., Luster M. I. (1995). Acetaminophen-Induced Hepatotoxicity Is Associated with Early Changes in NF-kB and NF-IL6 DNA Binding Activity. *Journal of Inflammation*.

[100] Tsujinaka T., Fujita J., Ebisui C. (1996). Interleukin 6 Receptor Antibody Inhibits Muscle Atrophy and Modulates Proteolytic Systems in Interleukin 6 Transgenic Mice. *Journal of Clinical Investigation*.

[101] Xie F., Fang C., Schnittke N., Schwob J. E., Ding X. (2013). Mechanisms of Permanent Loss of Olfactory Receptor Neurons Induced by the Herbicide 2,6-Dichlorobenzonitrile: Effects on Stem Cells and Noninvolvement of Acute Induction of the Inflammatory Cytokine IL-6. *Toxicology and Applied Pharmacology*.

[102] Smith T. D., Bhatnagar K. P. (2019). Anatomy of the Olfactory System. *Handbook of Clinical Neurology*.

[103] Su C. Y., Menuz K., Carlson J. R. (2009). Olfactory Perception: Receptors, Cells, and Circuits. *Cell*.

[104] Eichenbaum H., Otto T., Doty R. L., Müller-Schwarze D. (1992). The Hippocampus and the Sense of Smell. *Chemical Signals in Vertebrates 6*.

[105] Su J. H., Luo M. Y., Liang N. (2021). Interleukin-6: A Novel Target for Cardio-Cerebrovascular Diseases. *Frontiers in Pharmacology*.

[106] Dibattista M., Reisert J. (2016). The Odorant Receptor-Dependent Role of Olfactory Marker Protein in Olfactory Receptor Neurons. *Journal of Neuroscience*.

[107] Brann J. H., Firestein S. J. (2014). A Lifetime of Neurogenesis in the Olfactory System. *Frontiers in Neuroscience*.

[108] Turner J. H., May L., Reed R. R., Lane A. P. (2010). Reversible Loss of Neuronal Marker Protein Expression in a Transgenic Mouse Model for Sinusitis-Associated Olfactory Dysfunction. *American Journal of Rhinology & Allergy*.

[109] Zhang C., Zhang Y., Shen Y., Zhao G., Xie Z., Dong Y. (2017). Anesthesia/Surgery Induces Cognitive Impairment in Female Alzheimer’s Disease Transgenic Mice. *Journal of Alzheimers Disease*.

[110] Chen M., Reed R. R., Lane A. P. (2017). Acute Inflammation Regulates Neuroregeneration through the NF-*κ*B Pathway in Olfactory Epithelium. *Proceedings of the National Academy of Sciences of the United States of America*.

[111] Loddick S. A., Turnbull A. V., Rothwell N. J. (1998). Cerebral Interleukin-6 Is Neuroprotective during Permanent Focal Cerebral Ischemia in the Rat. *Journal of Cerebral Blood Flow & Metabolism*.

[112] Hirota H., Kiyama H., Kishimoto T., Taga T. (1996). Accelerated Nerve Regeneration in Mice by Upregulated Expression of Interleukin (IL) 6 and IL-6 Receptor after Trauma. *Journal of Experimental Medicine*.

[113] Sun L., Li Y., Jia X. (2017). Neuroprotection by IFN-*γ* via Astrocyte-Secreted IL-6 in Acute Neuroinflammation. *Oncotarget*.

[114] Zhang Y., Li L., Li T. (2018). In Vivo Measurement of the Dynamics of Norepinephrine in an Olfactory Bulb following Ischemia-Induced Olfactory Dysfunction and Its Responses to Dexamethasone Treatment. *Analyst*.

[115] Hummel T., Whitcroft K. L., Rueter G., Haehner A. (2017). Intranasal Vitamin A Is Beneficial in Post-Infectious Olfactory Loss. *European Archives of Oto-Rhino-Laryngology*.

[116] Guo Y. C., Yao L. Y., Wei Y. X. (2017). [Clinical Treatment Effect of Glucocorticoids and Extract of Ginkgo Biloba on Post-Viral Olfactory Dysfunction]. *Lin Chuang Er Bi Yan Hou Tou Jing Wai Ke Za Zhi*.

[117] Patel Z. M. (2017). The Evidence for Olfactory Training in Treating Patients with Olfactory Loss. *Current Opinion in Otolaryngology & Head and Neck Surgery*.

[118] Hummel T., Rissom K., Reden J., Hähner A., Weidenbecher M., Hüttenbrink K. B. (2009). Effects of Olfactory Training in Patients with Olfactory Loss. *Laryngoscope*.

[119] Dong Xing H. Q. Wei (2022). Research Progress on Olfactory Training for the Treatment of Olfactory Disorders. *Chinese Journal of Integrated Traditional Chinese and Western Medicine in Otolaryngology*.

[120] Liu S., Jiang Z., Zhao J. (2023). Disparity of Smell Tests in Alzheimer’s Disease and Other Neurodegenerative Disorders: A Systematic Review and Meta-Analysis. *Frontiers in Aging Neuroscience*.

[121] Doty R. L., Shaman P., Dann M. (1984). Development of the University of Pennsylvania Smell Identification Test: A Standardized Microencapsulated Test of Olfactory Function. *Physiology & Behavior*.

[122] Biggioggero M., Crotti C., Becciolini A., Favalli E. G. (2018). Tocilizumab in the Treatment of Rheumatoid Arthritis: An Evidence-Based Review and Patient Selection. *Drug Design, Development and Therapy*.

[123] Jiang S. J., Liang S. R., Zhang X. Q., Yang X. M. (2019). XM Yang. Research Progress on IL-6/IL-6 Receptor Axis and Its Targeted Inhibitors. *Chinese Journal of Cellular and Molecular Immunology*.

[124] Doty R. L., Kamath V. (2014). The Influences of Age on Olfaction: A Review. *Frontiers in Psychology*.

